# Supplementary material for: Development and Validation of a Ferroptosis-Related Gene Signature and Nomogram for Predicting the Prognosis of Esophageal Squamous Cell Carcinoma
Source: Front Genet. 2021 Oct 26;12:697524. doi: 10.3389/fgene.2021.697524 (PMC8576261; doi:10.3389/fgene.2021.697524)
Supplement: Supplementary file 1 [file Table1.DOCX]

Supplementary Table 1. Summary of ferroptosis-related genes.

| ABCC1 | ATP6V1G2 | DRD4 | GLS2 |
| --- | --- | --- | --- |
| ABHD12 | ATRA | DRD5 | GLUD1 |
| ACACB | AURKA | DUOX1 | GLUT13 |
| ACO1 | BACH1 | DUOX2 | GOT1 |
| ACSF2 | BAP1 | DUSP1 | GPT2 |
| ACSL3 | BECN1 | EGFR | GPX2 |
| ACSL4 | BID | EGLN1 | GPX4 |
| ACVR1B | BLOC1S5-TXNDC5 | EGLN2 | HAMP |
| AGPAT3 | BNIP3 | EHMT2 | HBA1 |
| AHCYL1 | BRD4 | EIF2AK2 | HDDC3 |
| AIFM1 | CA9 | EIF2AK3 | HELLS |
| AIFM2 | CAPG | EIF2AK4 | HERPUD1 |
| AKR1C1 | CARS1 | EIF2S1 | HIC1 |
| AKR1C2 | CAV1 | EIF4A1 | HIF1A |
| AKR1C3 | CBS | ELAVL1 | HILPDA |
| ALB | CCL5 | EMC2 | HMGB1 |
| ALDH3A1 | CD44 | EMP1 | HMGCR |
| ALDH3A2 | CDH1 | ENPP2 | HMOX1 |
| ALOX12 | CDKN1A | EPAS1 | HNF4A |
| ALOX12B | CDKN2A | ERN1 | HNRNPA1 |
| ALOX15 | CDO1 | FADS2 | HRAS |
| ALOX15B | CEBPG | FANCD2 | HSC70 |
| ALOX5 | CGAS | FBXL5 | HSD17B11 |
| ALOXE3 | CHAC1 | FBXW7 | HSF1 |
| ANGPTL4 | CHMP5 | FDFT1 | HSP90 |
| ANGPTL7 | CHMP6 | Fer1HCH | HSPA5 |
| ANO6 | CISD1 | FH | HSPB1 |
| ARF | CISD2 | FLT3 | IDH1 |
| ARF6 | COQ2 | FOXM1 | IDH2 |
| ARNTL | CP | FTH1 | IDO1 |
| ARRDC3 | CS | FTL | IFNG |
| ASNS | CTSB | FTMT | IL33 |
| ATF3 | CXCL2 | FXN | IL6 |
| ATF4 | CXXC1 | G6PD | IREB2 |
| ATG13 | CYBB | G6PDX | ISCU |
| ATG16L1 | D2HGDH | GABARAPL1 | ITGA6 |
| ATG3 | DDIT3 | GABARAPL2 | ITGB4 |
| ATG4D | DDIT4 | GABPB1 | ITGB8 |
| ATG5 | DECR1 | GCH1 | JDP2 |
| ATG7 | DLD | GCLC | JUN |
| ATM | DNAJB6 | GDF15 | KDM3B |
| ATP5MC3 | DPP4 | GFER | KEAP1 |
| KIM-1 | MTOR | PRDX1 | SMPD1 |
| KLHL24 | MUC1 | PRDX5 | SNORA16A |
| KRAS | MYB | PRDX6 | SNX4 |
| LAMP2 | MYC | PRKAA1 | SOCS1 |
| LATS1 | NAA38 | PRKAA2 | SOD2 |
| LATS2 | NADK | PRKCA | SP1 |
| LINC00336 | NCF2 | PROM2 | SQSTM1 |
| LINC00472 | NCOA3 | PSAT1 | SRC |
| LOC284561 | NCOA4 | PTGS2 | SRXN1 |
| LOC390705 | NEDD4 | RAB7A | STAT3 |
| LONP1 | NF2 | RB1 | STEAP3 |
| LPCAT3 | NFE2L2 | RELA | STING1 |
| LPIN1 | NFS1 | RGS4 | STMN1 |
| LURAP1L | NGB | RIPK1 | STYK1 |
| MAFG | NNMT | RNF113A | TAZ |
| MAP1LC3A | NOS2 | RNF20 | TF |
| MAP1LC3B | NOX1 | RPL8 | TFAM |
| MAP3K5 | NOX2 | RRM2 | TFAP2A |
| MAPK1 | NOX3 | SAHH | TFAP2C |
| MAPK14 | NOX4 | SAT1 | TFR2 |
| MAPK3 | NOX5 | SCD | TFRC |
| MAPK8 | NQO1 | SCP2 | TGFBR1 |
| MAPK9 | NR5A2 | SELENOS | TLR4 |
| MAPT | NRAS | SESN2 | TMBIM4 |
| MDM2 | OTUB1 | SETD1B | TNFAIP3 |
| MDMX | OXSR1 | SIAH2 | TP53 |
| MFN2 | PANX1 | SIRT1 | TP63 |
| MIF | PANX2 | SLC11A2 | TPD52 |
| MIOX | PARK7 | SLC1A4 | TRIB3 |
| MIR137 | PAX3 | SLC1A5 | TSC22D3 |
| MIR17 | PCBP1 | SLC2A1 | TUBE1 |
| MIR212 | PCK2 | SLC2A12 | TXN |
| MIR30B | PEBP1 | SLC2A14 | TXNIP |
| MIR4715 | PGD | SLC2A3 | TXNRD1 |
| MIR6852 | PHKG2 | SLC2A4 | UBC |
| MIR9-1 | PIK3CA | SLC2A6 | ULK1 |
| MIR9-2 | PLIN2 | SLC2A8 | ULK2 |
| MIR9-3 | PLIN4 | SLC38A1 | USP7 |
| MPO | PLP1 | SLC3A2 | VDAC1 |
| MT1G | PML | SLC40A1 | VDAC2 |
| MT3 | POLG | SLC7A11 | VDAC3 |
| MTDH | POR | SLC7A5 | VDR |
| MTF1 | PRC1 | SMAD3 | VEGFA |
| VHL | WIPI1 | YWHAE | ZFP36 |
| VLDLR | WIPI2 | YY1AP1 | ZFP69B |
| WDR76 | XBP1 | ZEB1 | ZNF419 |
| VHL | WIPI1 | YWHAE | ZFP36 |
